# The Swiss Light Source and SwissFEL at the Paul Scherrer Institute

**DOI:** 10.1140/epjp/s13360-023-03721-y

**Published:** 2023-02-06

**Authors:** Frithjof Nolting, Christoph Bostedt, Thomas Schietinger, Hans Braun

**Affiliations:** Paul Scherrer Institut (PSI), 5232 Villigen, Switzerland

## Abstract

At the Paul Scherrer Institute, two electron accelerator-based photon sources are in operation, namely a synchrotron source, the swiss light source (SLS), and an X-ray free-electron laser, SwissFEL. SLS has been operational since 2001 and SwissFEL since 2017. In this time, unique and world-leading scientific programs and methods have developed from the SLS and the SwissFEL in fields as diverse as macromolecular biology, chemical and physical sciences, imaging, and the electronic structure and behaviour of novel and complex materials. To continue the success, a major upgrade of SLS, the SLS2.0 project, is ongoing and at SwissFEL further endstations are under construction.

## Introduction to the facility: SLS and SwissFEL

The Paul Scherrer Institute (PSI) is the largest research centre for natural sciences and engineering in Switzerland and is part of the ETH Domain. The ETH Domain comprises the two Swiss Federal Institutes of Technology in Zurich (ETH Zurich) and Lausanne (EPFL), as well as four research institutes: the Swiss Federal Institute for Forest, Snow and Landscape Research (WSL), the Swiss Federal Laboratories for Materials Science and Technology (Empa), the Swiss Federal Institute of Aquatic Science and Technology (Eawag), and the Paul Scherrer Institute (PSI). PSI carries out excellent research in the fields of matter and materials, energy and the environment, and human health. By carrying out both fundamental and applied research, PSI has been contributing since 1988 to sustainable solutions for central questions arising within society, the economy, and science.

PSI develops, builds, and operates large-scale research infrastructures that are unique in Switzerland—and in some cases worldwide—including the Swiss Spallation Neutron Source (SINQ), the Swiss Light Source (SLS), the Swiss Muon Source (SμS), and SwissFEL, an X-ray Free-Electron Laser. These facilities are open to the user community via a proposal-based system; each year, there are around 4600 user visits. In addition to its research facilities, PSI operates Switzerland’s sole proton therapy centre for the treatment of specific malignant tumours.

The SLS has been a highly attractive research tool for many reasons. The excellent performance of the underpinning electron accelerator and storage ring, including its high performance reliability and stability [1–3], made the SLS the benchmark in synchrotron machine performance until well into the second decade of this century. With the advent of novel technologies in accelerator physics and the consequent emergence of the next generation of storage-ring facilities [4], known as diffraction-limited storage rings (or DLSRs), it has now become imperative to upgrade the SLS in like manner.

SwissFEL commenced photon science operations in late 2017 with two endstations at the Aramis hard X-ray branch [5]. After a period of commissioning and early science experiments, user operations started in 2019. Within one year, first light was generated in the Athos soft x-ray branch which can run in parallel of the hard X-ray branch at full repetition rate [6]. Over the past years, SwissFEL has reliably delivered intense X-ray pulses for a broad range of scientific applications of the biology, chemistry, physics, as well as materials sciences community. At the same time, novel beam modes and applications have been developed from the innovative and flexible SwissFEL accelerator design.

Complementary and in parallel to these developments in accelerator technology, huge progress in novel detector technologies at PSI [7] and the constant improvements at the beamlines at the SLS and SwissFEL have been driving the scientific success enabling higher spatial [8], temporal [9, 10] and spectral resolutions [11], and diverse sample environments for in-situ and operando studies [12]. It is expected that these developments will be significantly further driven by the upgrade of the SLS and the new endstations at SwissFEL. Last but not least, the scientific excellence, curiosity and drive for new scientific endeavors of the worldwide user community and the staff at PSI are a paramount pillar for the scientific impact of the facilities.

## Swiss light source

### Present status and scientific highlights

The Swiss Light Source (SLS) has been serving the international scientific community in endeavours as diverse as bioimaging, molecular biology, novel electronic materials, nanomagnetism, environmental science, catalysis and energy research, and cultural heritage, to name just some examples. Besides fundamental and applied research, the SLS is strong in industrial exploitation [13, 14], particularly by the pharmaceutical sector, but also more and more in materials science and advanced manufacturing applications. Among others, the SLS teams made key contributions to advanced method developments like scanning lensless imaging (ptychographic tomography), macromolecular crystallography, full-field tomography, soft X-ray angular-resolved photoelectron spectroscopy (SX-ARPES), and resonant inelastic X-ray scattering (RIXS). Besides the development of flexible sample environments, a specific focus has been the automation and remote control of the experiments, enabling groups to perform their research in hybrid teams with people both remotely connected and on site at PSI.

The interdisciplinary nature of PSI, centred on cutting-edge research facilities, and the significant prior investment in automation provided the basis for a fast response to the unexpected research challenges posed by COVID-19, where the SLS was able to contribute to many aspects of the underpinning science, ranging from structural biology [15] to pulmonary pathology [16]. The basic parameters of the SLS are given in Table 1.

**Table 1:** Parameters of the SLS [1, 2]

Energy	2.411 GeV
Lattice type	Triple bend achromat
Periodicity (arc geometry)	3
Circumference	288 m
Current (top up)	400 mA
Beam lifetime (average)	11.5 h
Horizontal emittance	5.59 nm rad
Vertical emittance	12 pm rad
Orbital tunes Qx, Qy	20.435/8.737
Momentum compaction factor	6.05 10^–4^
Main RF frequency	500 MHz
Peak RF voltage	2.6 MV
Third harmonic RF cavity	1500 MHz
Radiation loss/turn	512 keV
Energy spread (rms)	8.57 10^–4^
Energy stability	2 10^–5^
Damping times (x, y, E)	9, 9, 4.5 ms

### Technological developments and upgrade plans for the decade 2023–2033

In recent years, major advances in the design of synchrotrons and insertion devices have made possible an increase in the brightness of X-ray beams by substantial factors. In parallel, experimental methods, detector technology, data acquisition and processing capabilities have evolved, thus permitting one to exploit this increased photon-beam brightness for the benefit of higher spatial resolution, larger and smaller samples, and faster measurements at the beamlines. To maintain the SLS at the forefront of synchrotron user facilities, PSI prepared a major upgrade project for the SLS, named SLS 2.0 [17–21].

Key components of the SLS upgrade project are:

- A complete rebuild of the storage ring in the existing storage ring building using an innovative multibend achromat and antibend design for optimum beam brightness. Ring circumference (288 m), bunch time pattern (500 MHz) and stored beam current (400 mA) remain as before.
- Increase of the electron beam energy from 2.4 to 2.7 GeV to increase the photon energy range and flux.
- New undulator and superbend radiation sources optimally adapted to the needs of the beamlines.
- Coherent concepts of data acquisition and processing for the beamlines, well adapted to the increased performance of the X-ray source.
- Systematic exploitation of all opportunities for reduced power consumption, thus permitting long-term sustainable operation.

**Fig. 1:**
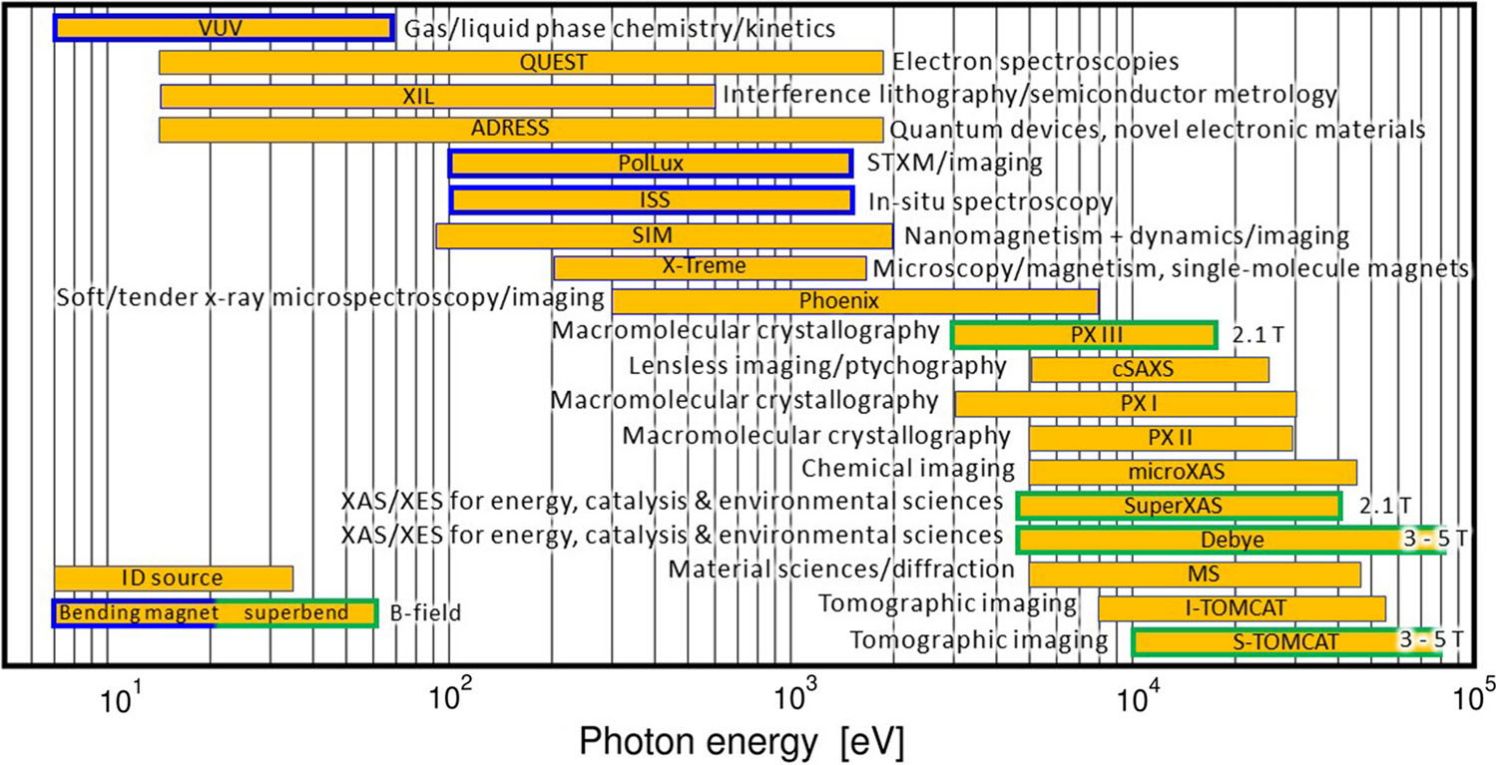
Overview of beamline in user operation after the upgrade. The source type (ID source, Bending and superbend together with magnetic field strength), the beamline acronym, major applications area and energy range are shown

The various measures will lead to very significant performance improvements. The key figure of merit for the electron beam, the horizontal emittance, is reduced by more than a factor 40. The improvement in photon-beam quality cannot be summarized in a single figure, since it depends strongly on the individual photon sources and photon energies. The main implementation phase, or “dark time”, to dismantle the existing storage ring and to install the new ring will be from October 2023 to December 2024. For resource reasons, the various beamline upgrades have been distributed in two phases and first pilot users for selected beamlines at SLS 2.0 are expected in the Summer/Fall of 2025, which will be followed by a further shutdown at the beginning of 2026. The beamlines of the second phase will become available for users in the second half of 2026, see Fig. 1 for an overview.

**Fig. 2:**
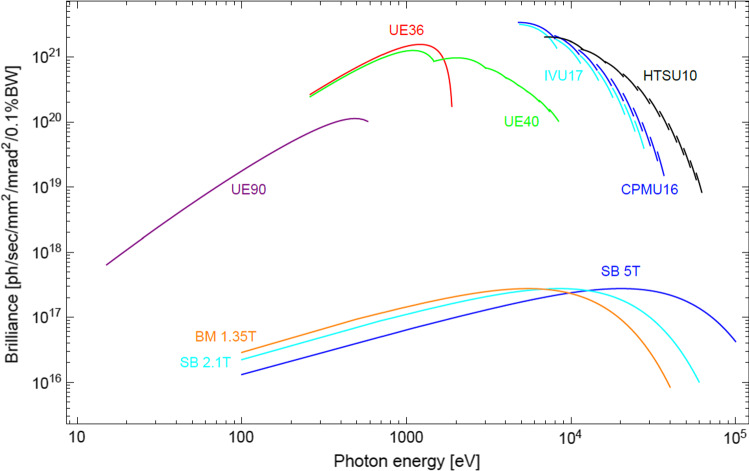
Brilliance curves of the SLS 2.0 sources. CPMU = cryogenic permanent-magnet undulator; HTSU = high-temperature superconducting undulator; UE = elliptical undulator; two-digit suffices = undulator periodicity in mm; IVU = In-Vacuum Undulator BM = bending magnet; SB = superbend; field strengths in Tesla

**Fig. 3:**
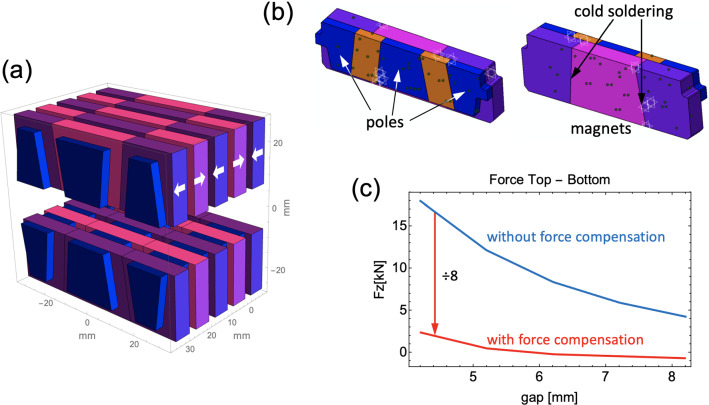
Novel developments in hard x-ray insertion devices. **a** The central Halbach array of poles and magnets can be made to be significantly narrower, thanks to an improved injection scheme with a (close to) on-axis injection. Consequently, the forces for a given central magnetic-field strength will be lower. Moreover, the central magnet array can be flanked by arrays in which the poles are opposed (N–N or S–S), thus reducing the total forces even more. The configuration is shown in **b**. The reduction in force is typically a factor of eight or more **c**, allowing for far more compact and inexpensive mechanical designs

 An exciting aspect of the upgrade is that improvements in the brilliance also enable other innovations further down the technological chain, notably in the field of undulator development, see Fig. 2 for an overview of the expected brilliances of the different insertion devices [22].^1^ The narrower electron beam presents several technological opportunities. Firstly, entire undulator maxima can be used for those experiments that do not require a very small relative bandwidth but require as many photons per unit time on the sample as possible. At SLS 2.0, these might include certain types of diffraction techniques such as serial crystallography, lensless imaging that relies primarily on the transverse (and not longitudinal) coherence, and imaging techniques such as phase-contrast tomography. Secondly, the reduced oscillations of the electron beam due to the improved injection scheme from the booster means that as it passes along the undulator, the width of the magnets needed to produce a homogeneous field across the central axis can be reduced by over a factor of two. This means that the forces acting on the undulator support structure become concomitantly smaller. Moreover, the additional space gained by making the magnets narrower also allows the incorporation of two additional sets of magnets on each side of the central array, which, in contrast to the central array, are poled so that they repel [23], see Fig. 3. This will reduce the net forces on the undulator frame by well over an order of magnitude compared to standard devices used today, and will likely make them more economic and much more compact and reliable. A further ambitious R&D project in the area of hard x-ray insertion devices is being pursued as part of the upgrade SLS program and integrated in the CHART and LEAPS Innovation program, namely the development of an ultra-short-period hard X-ray device (*λ*_u_ = 10 mm) in order to access photon energies in excess of 50 keV, despite the moderate storage-ring energy of SLS 2.0 of 2.7 GeV [24, 25]. High *K* values are required to enhance the intensities of higher-harmonic radiation, which in turn means that magnetic field strengths are required that can only be achieved with advanced high-temperature superconducting materials.

## SwissFEL

### Present status and scientific highlights: SwissFEL

**Fig. 4:**
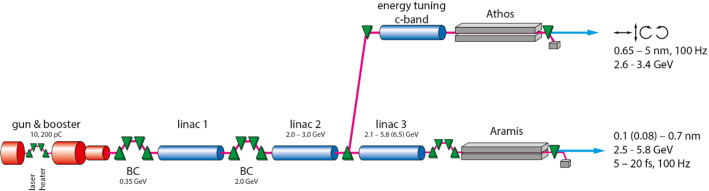
Layout of the SwissFEL facility

SwissFEL is currently running the Aramis hard X-ray and Athos soft X-ray lines in full parallel operations. The overall layout of the facility is depicted in Fig. 4. Two electron bunches are accelerated in the first two common linear accelerator (linac) sections in two radiofrequency buckets separated by 28 ns up to a beam energy of 3 GeV. Then a fast kicker separates the two bunches, the first one continues straight into a third linac section with a beam energy up to 6 GeV while the second electron bunch is diverted into the parallel soft X-ray line.

The Aramis branch features in-vacuum undulators that can deliver photon energies from the tender (approx. 2 keV) to the hard (approx. 12 keV) X-ray regime. The Athos soft X-ray branch has Apple-X undulators with full polarization control, delivering photon energies from approx. 0.25 keV up to 1.9 keV. Pulse energies at SwissFEL reach several mJ in the soft X-ray and exceed 1 mJ for most of the tender to hard X-ray regime for full bunch charge. SwissFEL can run in full parallel operations, delivering 100 Hz to each undulator branch. A variety of special beam modes is available that are described in detail in the following section.

Each of the branch lines can host up to three for a total of six experimental endstations. Currently the following three endstations are in user operations:

- Alvra: Hard X-ray endstation with a focus on photo-chemistry and -biology. Specific strength of the endstation for chemical applications include ultrafast absorption and emission spectroscopy in the tender to hard x-ray regime for molecular systems and nanoparticles in solution [26, 27]. For photobiology, a lipidic cubic phase (LCP) injector combined with a 16 megapixel Jungfrau pixel detector enables time-resolved serial femtosecond crystallography with time resolution of tens of femtoseconds and pump-probe delays ranging from the femtosecond to the millisecond scale [10].
- Bernina: Hard X-ray endstation for condensed matter physics and material sciences. Selective X-ray probes that are sensitive to electronic, magnetic, or atomic structures allow the investigation of ultrafast phenomena such as ultrafast electronic phase transitions [28], correlated materials in the time domain [29], and alike. The endstation also serves as a flexible platform for a variety of experiments ranging from developments for hard X-ray transient gratings [30] to supercooled states of water [31].
- Maloja: Soft X-ray endstation for atomic, molecular, optical physics as well as chemistry and non-linear X-ray sciences. The latest endstation commencing user operations in 2022 features a flexible setup in which a variety of spectroscopy, imaging, and sample delivery modules can be combined. Scientific applications range from light-matter interaction to light induced chemical reactions in the gas and liquid phase as well as to single particle imaging [32]. The endstation is also used for the characterization of new Athos pulse modes [33].

In addition, there are currently two endstations in commissioning with expected start of user operations in 2023:

- Cristallina: Hard X-ray endstation for ultrafast diffraction of quantum materials and biological systems
- Furka: Soft X-ray endstation for the study of correlated quantum materials with time-resolved resonant inelastic scattering, absorption, and diffraction.

Ultrafast optical laser systems are available for the endstations with secondary sources for the specific science needs ranging from THz to UV frequencies. A timing and synchronization system paired with X-ray optical cross correlators available at the endstations allow for time resolutions on the order of a few tens of femtoseconds.

### Technological developments and upgrade plans for the decade 2023–2033: SwissFEL

In the near future, the developments at SwissFEL will focus on enhanced accelerator modes enabled by the flexible Athos undulator line design. Compact chicanes between undulator segments in combination with the versatility of the APPLE-X undulators allow a reduction of the required undulator length to achieve FEL saturation, the improvement of the longitudinal coherence of the FEL pulses, and the ability to produce shorter FEL pulses with higher power levels [34].

To further improve the longitudinal coherence and spectral stability of Athos, an external laser seeding scheme is currently being installed and commissioned. In a first step, the laser beam was successfully overlapped with the electron bunch. The external laser system is used to seed the emission of X-ray photons, and thus imprint well-defined optical properties on the beam. In the next step, a second laser pulse will be used to implement the full Echo Enabled Harmonic Generation (EEHG) scheme, giving the Athos beamline excellent spectral and intensity stability and also longitudinal coherence [35, 36]. Concurrently, planning for the last remaining endstation is ongoing. The Diavolezza endstation will be designed to take unique advantage of the seeded pulses soon available in the Athos branch.

In the long term, towards the end of this decade, a third undulator line is planned [37]. The Porthos branch of SwissFEL is envisioned for very hard X-ray emission and will also include the flexible pulse generation concepts pioneered on Athos. Porthos will incorporate the most recent advances in accelerator and undulator technology. With the unique, modular undulator design at SwissFEL, new approaches will be explored in X-ray pulse shaping to generate attosecond X-ray pulses and pulse trains with continuously variable time structure, and the capability for multi-color and non-linear X-ray experiments will be exploited. Once established for soft X-rays, these methods will be adapted for hard X-rays at Porthos, enabling new experiments in the biological, chemical, materials, and physical sciences to address paradigm-shifting scientific challenges.

## Perspective of next scientific challenges

With these upgrades and the constant improvements of the beamlines and endstations, we are targeting three central ‘grand challenges’: human health, energy and the environment, and matter and materials. They address urgent societal demands in the twenty-first century, including clean and low-cost energy generation, delivery, and consumption; affordable, targeted, and personal medicine to cater to an ageing population; and high-capacity and energy-efficient information technology. All these drivers require an understanding of materials, devices, and biomedical systems on a nanoscale- or atomic level. Breakthroughs will depend intimately on the development of novel, tailor-made functional materials designed often at many spatially hierarchical levels down to the atomic scale. Besides progress in fundamental and applied research, a close link to technology transfer, enabling industry to take advantage of the developments, is essential. Such ambitious goals can, however, only be achieved if a palette of suitable research facilities and methods are available. The upgraded SLS and SwissFEL promise to play a central role in this endeavour.

## Footnotes

1 This paragraph is adapted from [22] with the permission of Ph. Willmott and the SSPS.

## Declarations

### Data availability

No data associated in the manuscript.
